# FOR BETTER AND WORSE? THE IMPORTANCE OF CLOSENESS AND AGE FOR SPOUSES’ CARDIOMETABOLIC SIMILARITY

**DOI:** 10.1093/geroni/igz038.1628

**Published:** 2019-11-08

**Authors:** Stephanie J Wilson, Juan Peng, Rebecca Andridge, Lisa M Jaremka, Christopher P Fagundes, William B Malarkey, Martha A Belury, Janice K Kiecolt-Glaser

**Affiliations:** 1 The Ohio State University College of Medicine, Columbus, Ohio, United States; 2 The Ohio state University, Columbus, Ohio, United States; 3 University of Delaware, Newark, Delaware, United States; 4 Rice University, Houston, Texas, United States; 5 The Ohio State College of Medicine, Columbus, Ohio, United States

## Abstract

Spouses share age-related disease risks: a person’s diabetes or hypertension raises the partner’s odds for the same condition. To probe the importance of partners’ closeness, marital satisfaction, and age for spouses’ similarity in cardiometabolic health, 43 disease-free couples ages 24-61 provided fasting glucose, fat and carbohydrate oxidation, and blood pressure at two study visits. Couples who felt closer had more similar rates of carbohydrate oxidation compared to those who felt less close. Likewise, happier couples had more similar carbohydrate and fat oxidation. Fasting glucose and blood pressure were more similar within middle-aged couples compared to younger pairs. In follow-up analyses, partners’ health behavior concordance did not explain these effects. In sum, closer, happier, and older couples shared more similar cardiometabolic profiles, perhaps driven by joint stress and emotional spillover. Findings suggest that closer, happier relationships may confer both larger health risks and benefits, and increasing age may raise the stakes.

